# An Aversive Response to Osmotic Upshift in *Caenorhabditis elegans*

**DOI:** 10.1523/ENEURO.0282-16.2017

**Published:** 2017-04-21

**Authors:** Jingyi Yu, Wenxing Yang, He Liu, Yingsong Hao, Yun Zhang

**Affiliations:** 1 Department of Organismic and Evolutionary Biology, Center for Brain Science, Harvard University, Cambridge, MA 02138; 2 Department of Molecular Biology, Massachusetts General Hospital, Harvard Medical School, Boston, MA 02114; 3 Department of Neurobiology, Harvard Medical School, Boston, MA 02114

**Keywords:** cGMP-gated channel, osmotic stimuli, sensorimotor response

## Abstract

Environmental osmolarity presents a common type of sensory stimulus to animals. While behavioral responses to osmotic changes are important for maintaining a stable intracellular osmolarity, the underlying mechanisms are not fully understood. In the natural habitat of *Caenorhabditis elegans*, changes in environmental osmolarity are commonplace. It is known that the nematode acutely avoids shocks of extremely high osmolarity. Here, we show that *C. elegans* also generates gradually increased aversion of mild upshifts in environmental osmolarity. Different from an acute avoidance of osmotic shocks that depends on the function of a transient receptor potential vanilloid channel, the slow aversion to osmotic upshifts requires the cGMP-gated sensory channel subunit TAX-2. TAX-2 acts in several sensory neurons that are exposed to body fluid to generate the aversive response through a motor network that underlies navigation. Osmotic upshifts activate the body cavity sensory neuron URX, which is known to induce aversion upon activation. Together, our results characterize the molecular and cellular mechanisms underlying a novel sensorimotor response to osmotic stimuli and reveal that *C. elegans* engages different behaviors and the underlying mechanisms to regulate responses to extracellular osmolarity.

## Significance Statement

Extracellular osmolarity presents an ecological factor that is essential for the survival of all organisms. However, how animals respond to environmental osmolarity is not fully understood. We show that *Caenorhabditis elegans* responds to the upshifts of extracellular osmolarity by gradually increasing reorienting movements, and the aversive response requires a conserved cGMP-gated channel subunit TAX-2. TAX-2 acts in several neurons that are exposed to the body fluid to regulate the response to osmotic upshifts. This behavior uses different channels and sensory neurons than those previously characterized in acute avoidance of osmotic shocks, which is mediated by a transient receptor potential vanilloid channel in a sensory neuron open to the outside. Thus, our results reveal a novel sensorimotor response to extracellular osmolarity and the underlying mechanisms.

## Introduction

A stable intracellular osmolarity is essential for survival. Both hyperosmotic and hypoosmotic conditions, when the extracellular osmolarity is higher and lower than the intracellular osmolarity, respectively, alter the water content, the volume, and the ionic concentration of a cell. These perturbations often change the activity of the intracellular proteins and nucleotides, resulting in detrimental effects on the physiology and the function of a cell (Steenbergen et al., 1985; Somero, 1986; Garner and Burg, 1994). Many organisms, including bacteria, yeast, plants and animals, use physiologic and behavioral strategies to reduce the intracellular perturbations generated by osmotic stimuli.

The physiologic response to hyperosmolarity has been well studied in yeast, where osmotic upshifts induce the biosynthesis of glycerol that functions as an osmolyte to increase intracellular osmolarity (Hohmann, 2002). In contrast, mammals regulate homeostatic responses, such as urine production, to hyperosmotic and hypoosmotic conditions to maintain a stable internal osmolarity (Thrasher et al., 1980a,b; Zerbe and Robertson, 1983). Meanwhile, free-living animals also behaviorally respond to osmotic stimuli. For example, a ventricular infusion of a hyperosmotic solution in dogs or an intravenous infusion of a hyperosmotic solution in humans increased water intake (Thrasher et al., 1980b; Zerbe and Robertson, 1983). Several brain areas become active shortly after an infusion of a hyperosmotic solution, and electrical stimulation of some of these brain regions, including the anterior cingulate area, induces drinking in monkeys (Egan et al., 2003; Sewards and Sewards, 2003). Some neurons in the organum vasculosum laminae terminalis, the supraoptic nucleus, or the paraventricular nucleus respond to changes in the extracellular osmolarity and are proposed to function as the central osmoreceptor neurons (Oliet and Bourque, 1992, 1993; Qiu et al., 2004; Ciura and Bourque, 2006).

At the molecular level, members of the mammalian transient receptor potential vanilloid (TRPV) channels have been characterized for their osmosensory functions. TRPV1 is expressed in the central osmosensory neurons and the Trpv1 knock-out mice lose the response to hyperosmolarity in these neurons (Ciura and Bourque, 2006; Sharif Naeini et al., 2006). TRPV4 is also expressed in the osmosensory brain regions, and the Trpv4 knock-out mice display defective physiologic and behavioral responses to osmotic stimuli (Liedtke et al., 2000; Liedtke and Friedman, 2003). The model organism *Caenorhabditis elegans* encodes several homologs of the mammalian TRPV channels, one of which is OSM-9, which acts in a polymodal sensory neuron ASH to detect strong osmotic shocks that induce acute avoidance. Expression of the mammalian TRPV4 in ASH substitutes for the function of *osm-9* in directing avoidance of osmotic shocks (Colbert et al., 1997; Liedtke et al., 2003).

While these previous studies provide substantial understanding of osmosensory response, the underlying mechanisms are not fully addressed. *C. elegans* provides an opportunity to investigate this question. The nematode is often found in rotten organic materials, a heterogeneous environment that presents osmotic stimuli in various ways. It is known that *C. elegans* acutely avoids high osmolarity by generating reversals immediately on a direct contact. This response is mediated by a homolog of the mammalian TRPV channels in the sensory neuron ASH that is open to the outside (Colbert et al., 1997; Hilliard et al., 2004, 2005). Here, we show that *C. elegans* also displays an aversive response to mild osmotic upshifts when it exposes the body to the upshift for a few minutes. However, different from the avoidance of the high osmolarity, the gradually increased aversion of the osmotic upshifts requires a cGMP-gated channel subunit, TAX-2, which acts in a set of body cavity sensory neurons that are exposed to the body fluid. Osmotic upshifts activate one of the body cavity neurons, URX, which is known to induce aversion when activated. The aversive response evoked by osmotic upshifts depends on a sensorimotor circuit that regulates reversals and turns. Together, our results define a new type of behavioral response to osmotic stimuli and elucidate the underlying molecular and circuit mechanisms.

## Materials and Methods

### Strains

All strains were cultivated under the standard conditions (Brenner, 1974). The strains used in this work include: wild-type (WT) strain Bristol N2, ZC2493 *tax-2(p691)*I, PR671 *tax-2(p671)*I, am1 *osr-1(rm1)*I, BE93 *dpy-2(e8)*II, ZC2533 *tax-2(p691)*I; *yxEx1291[tax-2 genomic pcr; Punc-122::gfp]*, ZC2547 *tax-2(p671)*I; *yxEx1315[tax-2 genomic fusion pcr; Punc-122::gfp]*, ZC2691 *tax-2(p691)*I; *yxEx1325[Pgcy-36::tax-2cdna; Punc-122::rfp]*, ZC2407 *yxEx1257[Pttx-3::twk-18(gf)::mcherry; Podr-2b(3a)::twk-18(gf)::mcherry; Punc-122::gfp]*, ZC2408 *yxEx1258[Pttx-3::twk-18(gf)::mcherry; Pinx-1::twk-18(gf)::mcherry; Punc-122::gfp]*, ZC2406 *yxEx1256[Pinx-1::twk-18(gf)::mcherry; Punc-122::rfp]*, ZC2393 *yxEx1248[Pttx-3::twk-18(gf)::mcherry; Punc-122::rfp]*, ZC1552 *yxEx778[Pglr-1::tetx::mCherry; Punc-122::gfp]*, CX7102 *lin-15(n765)X; qaIs2241*[*Pgcy-36::egl-1*; *Pgcy-35::gfp*; *lin-15(*+)]X.

### Generation of transgenes

The rescuing *tax-2* genomic region contained the *tax-2* coding region, 2.3 kb 5' sequence and 400 bp 3' sequence. Two PCR fragments with an overlapping sequence of 500 bp were generated to cover the entire *tax-2* genomic sequence and were injected together into the *tax-2(p691)* animals at 10 ng/µl each. A PCR product that was generated from a fusion PCR from the above two overlapping fragments and contained the same genomic region was injected into the *tax-2(p671)* animals at 5 ng/µl. The primers used in these experiment are as follows: forward primer, CTTGAGCCTCTCGACCCATACC; reverse primer, CACATACTGAGCCTGCACTG; and the additional primers for the overlapping regions, GATCTGACAATCGTCGCCAGTG, and TGGGCTACTTCGAGCATTTCTG.

To generate the *Pgcy-36::tax-2* construct or the *Pstr-3::tax-2* construct, a PCR product of the *gcy-36* promoter (forward primer, CATGATGTTGGTAGATGGGGTTTG; reverse primer, ATTGTTGGGTAGCCC TTGTTTGA) or the *str-3* promoter (forward primer, TGGTGAAGA TTTGTTCAAGGACG; reverse primer, GTTCCTTTTGAAATTGAGGCAGTTG) was cloned into the Invitrogen pCR 8 TOPO TA vector to make an entry clone. The PCR product of *tax-2* cDNA (forward primer, ATGGTACCATGTATCAAGTTCCAAAACGAG; reverse primer, ATGGTACCTTAATCGGCATGTAGTTTCTGTG) was introduced into a destination vector derived from pPD95.77. LR recombination (Invitrogen) between the *Pgcy-36* entry clone or the *Pstr-3* entry clone and the *tax-2* cDNA destination clone were used to generate *Pgcy-36::tax-2* or *Pstr-3::tax-2*, respectively. Each of the constructs was injected into the *tax-2(p691)* mutant animals at 5 ng/µl to generate transgenic lines.

### Solution preparation

*C. elegans* responds to NaCl; therefore, to control for the behavioral response to NaCl, all of the solutions used in the study had the same concentration of NaCl at 50 mm. Specific osmolarity was generated with glycerol, NaCl, or sorbitol. Glycerol was used when the solute was not specifically described. The final osmolarity of each solution was calculated by adding up the osmolarity contributed from each solute in the solution. We did not consider the effects on osmolarity that were generated by potential interactions among solutes, which were usually small (see the chart below). The osmolarity from each solute is the total number of elements the solute contributes when fully dissolved in water. Assuming that NaCl is fully ionized at the concentrations used, 1 mm NaCl contributes to 2 mOsm, and 1 mm glycerol or sorbitol contributes to 1 mOsm. The calculation was validated by measuring the osmolarity of each solution with an osmometer (Vapro Vapor Pressure Osmometer 5520), as shown in the following chart.

The osmolarity of the standard nematode growth medium (NGM) was measured to be 158 mOsm.

**Table:** Preparation o*f* testing solutions

Balancing solute	Calculated osmolarity (mOsm)	Measured osmolarity (mOsm)
Glycerol	150	144
	400	415
	600	621
	800	838
NaCl	150	144
	400	369
Sorbitol	150	141
	400	373

### Droplet assay

The droplet assay was performed essentially as described with modifications (Luo et al., 2008; Ha et al., 2010). In each assay, four droplets of a 6 μl solution were placed on a sapphire window (Swiss Jewel Company). Adult worms were collected from cultivating food plates, washed in NGM buffer for 10 min to eliminate the turning response to food removal, and then individually transferred with a small platinum wire into the droplets. One adult worm was placed into each droplet. The movement of the worms was recorded by a CCD camera at 10 Hz. The temperature of the sapphire window was kept at 23°C using a temperature-controlled water bath. The recorded movement was analyzed by machine vision software written in MATLAB. During swimming, *C. elegans* generated continuous body thrashes that were interrupted by reversals and big body bends that resembled the shape of Ω. We categorized both reversals and big body bends as turns. Specifically, to identify turns, the MATLAB code fitted the image of a worm in each frame into an ellipse and calculated the eccentricity of the ellipse. The eccentricity was smaller during turns, because the shape of the worm was more circular. A turn was defined to occur when the eccentricity was smaller than a threshold value, which was previously determined with manually detected turns. We measured the swimming speed by quantifying the number of body thrashes per minute. We counted the curving of a worm body toward either side as one thrash. We also measured the volume of the four droplets before and after the assay for multiple assays and found no significant difference (*V*_before_ = 23.6 ± 0.31 µl; *V*_after_ = 23.2 ± 0.35 µl; *p* = 0.38, Student’s *t* test, *n* = 8; mean ± SEM). Wild-type control and comparative genotypes were tested sequentially on the same day in the same experiment.

### Measurement of dehydration

Worms were cultured on regular NGM plates (∼150 mOsm). To measure the dehydration caused by different osmotic conditions, a worm was first transferred onto an agar plate that had the same osmolarity as the cultivating plate to take pictures with an Eclipse TE2000U microscope with a CoolSNAP EZ CCD camera (Nikon). The worm was then soaked in a 200 or 400 mOsm solution for 8 min and returned to the original agar plate to take another set of pictures. Three pictures were taken for each worm under each condition. The outline of each worm was manually traced, and the area of the worm body was measured with the NIS-element software (Nikon) in pixels. To minimize the subjective bias during the measurement, the picture files were randomly ordered and renamed using a MATLAB code. The measurement was performed with the researcher blind to the conditions.

### Adaptation assay

NGM plates with osmolarity of 400 mOsm were made by adding sorbitol to the mixture of the regular NGM plates, which had osmolarity of 150 mOsm. To adapt animals to the hyperosmotic conditions, wild-type worms were cultivated on the 400 mOsm NGM plates for one generation before testing. During the assay, adapted adults were soaked for 10 min in a 400 mOsm solution balanced by glycerol and then transferred to droplets of the 200 or 400 mOsm solution to record behavior. Control animals were cultivated on regular NGM plates, soaked in the regular NGM buffer for 10 min, and tested in droplets of the 200 and 400 mOsm solution in parallel.

### Calcium imaging

Calcium imaging and analysis were performed essentially as previously described (Chronis et al., 2007; Ha et al., 2010). The GCaMP6 signal in the cell body of the URX neuron was measured using ImageJ (NIH). The average GCaMP6 signal for the first 60 s in each recording was used as baseline (*F*_base_) to calculate the change in GCaMP6 signal (Δ*F*) for every fame for the first 120 s recording for each animal [Δ*F* = (*F* − *F*_base_)/*F*_base_ * 100%]. The bleaching effect was corrected by the built-in function “Bleach Correction” of Fiji (Schindelin et al., 2012) using the exponential fit. The calcium imaging data are normally distributed examined with either the Kolmogorov–Smirnov test or the Shapiro–Wilk test, and the difference between the responses to high and low osmolarity is tested with the Student’s *t* test.

## Results

### *C. elegans* aversively responds to osmotic upshifts

**Figure 1.**
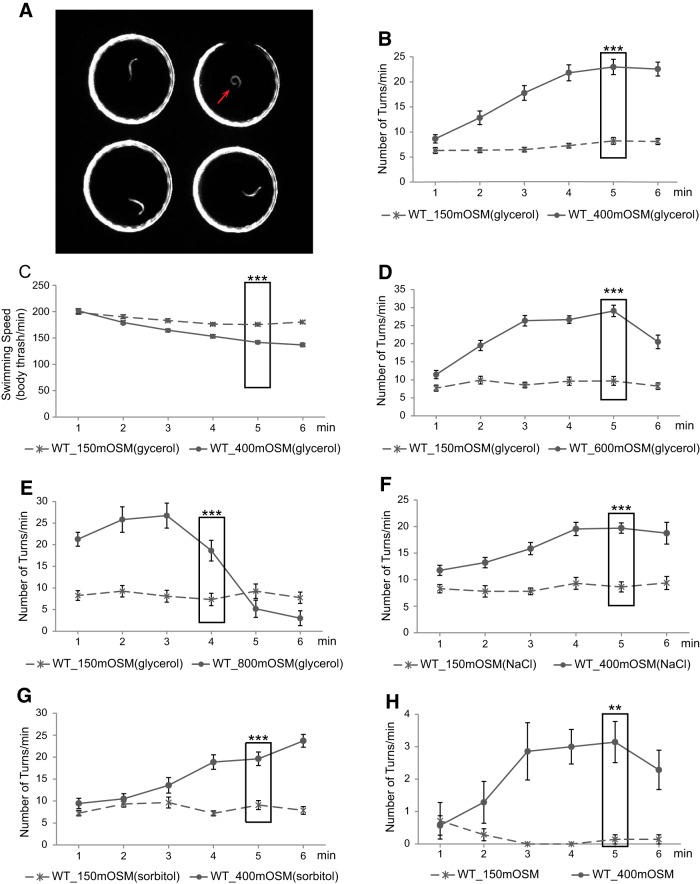
*C. elegans* generates an aversive response to mild osmotic upshifts. ***A***, A sample image of the droplet assay (Materials and Methods). Each 6 µl droplet of buffer contains one worm, and the arrow denotes a large body bend. ***B***, WT animals that are cultivated under the standard osmotic condition (∼150 mOsm) display a stable turning rate in a glycerol solution of 150 mOsm but gradually and significantly increase the turning rate in a hyperosmotic glycerol solution of 400 mOsm. *n* = 55 each. ***C***, WT animals that are cultivated under the standard osmotic conditions (∼150 mOsm) display a significantly lower thrashing speed in a hyperosmotic glycerol solutions of 400 mOsm than in a glycerol solution of 150 mOsm. *n* = 55 each. ***D***, ***E***, WT animals that are cultivated under the standard osmotic condition (∼150 mOsm) also gradually and significantly increase the turning rate in the hyperosmotic glycerol solutions of 600 mOsm (***D***; *n* = 27 each) or 800 mOsm (***E***; *n* = 12 each). Note that in ***E*** the turning rate starts to drop after 3 min in the 800 mOsm solution, due to decreased mobility of the animals possibly caused by server dehydration. Therefore, the statistical test is performed on the turning rates in the 4th minute in ***E***. ***F***, ***G***, WT animals cultivated under the standard osmotic condition (∼150 mOsm) display an increased turning rate in a 400 mOsm hyperosmotic solution that is balanced by either NaCl (***F***; *n* = 24 each) or sorbitol (***G***; *n* = 24 each) in comparison with the turning rate in the 150 mOsm solution. For ***B****–****G***, the turning rates in solutions of different osmolarities in the 5th minute (in the 4th minute in ***E***) are compared using the Student’s *t* test. ***H***, WT animals that are cultivated under the standard osmotic condition (∼150 mOsm) display a stable turning rate on a NGM plate of 150 mOsm but gradually and significantly increase the turning rate on a NGM plate of 400 mOsm. The osmolarity of the plates is balanced by sorbitol. The turning rates on plates of different osmolarity are compared using the Student’s *t* test. *n* = 7 animals each. For all, ***p < 0.001, **p < 0.01. Values are reported as the mean ± SEM. WT, wild type.

The osmolarity of the standard culturing medium for *C. elegans* is ∼150 mOsm. Exposure to solutions of ≥1 Osm osmolarity induces shrinkage of the worm body and immobilization in a few minutes. A long-term exposure to similar osmolarity induces adaption and resistance due to increased accumulation of glycerol in the body (Lamitina et al., 2004; Solomon et al., 2004). *C. elegans* acutely avoids solutions of high osmolarity (≥1 Osm) by generating reversals within a few seconds of a direct encounter, and the avoidance requires the *C. elegans* homolog of the mammalian TRPV channels in the sensory neuron ASH. In contrast, on direct contact *C. elegans* does not generate a similar reversing response to hyperosmotic stimuli ranging from 200 mOsm to 1 Osm (Colbert et al., 1997; Liedtke et al., 2003; Hilliard et al., 2004, 2005). Because solutions that have osmolarity within this range also cause shrinkage of the body within a few minutes of exposure and induce a long-term adaption (Liedtke et al., 2003; Solomon et al., 2004), we examined whether *C. elegans* displayed a different behavioral response to hyperosmotic stimuli of this range. We used an automated droplet assay that allowed the nematode to swim in 6 µl drops of a solution and measured the rate of reversals and large body bends (Fig. 1*A*; Luo et al., 2008; Ha et al., 2010). Previously, we have shown that during swimming *C. elegans* generates continuous “C-shaped” body swings that are occasionally interrupted by turns that include reversals and large body bends and are followed by reorienting movements. Attractive stimuli suppress the turning rate, while removal of attractive stimuli or exposure to repulsive cues increases it (Bargmann et al., 1993; Clark et al., 2007; Luo et al., 2008; Srivastava et al., 2009; Ha et al., 2010). Therefore, an increase in the number of turns within a given amount of time represents an aversive response (Fig. 1*A*). We found that the worms cultivated on the standard NGM, which had an osmolarity of ∼150 mOsm, displayed a stable turning rate in a glycerol solution of 150 mOsm. In comparison, when exposed to a glycerol solution of 400 mOsm these animals gradually and significantly increased their turning rate and reached the highest turning rate after 5 min (Fig. 1*B*). It is conceivable that the worms generated the behavioral response as part of the response to dehydration. Consistently, we found that the worms that were cultivated under the condition of 150 mOsm shrank their bodies to (mean ± SEM) 97.5% ± 1.1% or 77.6% ± 2.1% (*n* = 7 each), respectively, of the area that their bodies occupied on a flat image after swimming in a glycerol solution of 200 or 400 mOsm for 8 min (Materials and Methods). In addition, we measured how fast the worms thrashed their bodies in the 150 and 400 mOsm solutions (Materials and Methods) and found that they thrashed significantly less in the 400 mOsm glycerol solution (Fig. 1*C*), suggesting that the increase in the turning rate does not result from a general increase in locomotion. We also examined the behavioral response to glycerol solutions of higher osmolarity, 600 and 800 mOsm, and found that both stimuli increased the turning rate (Fig. 1*D*,*E*) within a few minutes after exposure. To address whether the aversive response was induced by an increase in osmolarity or by an increase in the concentration of glycerol, we examined the animals that were exposed to a NaCl solution or a sorbitol solution of similar osmolarity. We found that exposure to either a NaCl solution of 400 mOsm or a sorbitol solution of 400 mOsm similarly induced an increase in the turning rate (Fig. 1*F*,*G*). Together, these results indicate that with a continuous exposure *C. elegans* aversively responds to osmotic upshifts by gradually increasing reorienting movements. For the rest of the study, we used glycerol solutions to characterize the behavioral response to osmotic upshifts.

Next, we asked whether *C. elegans* responded to osmotic upshifts on a solid substrate in a manner similar to that in a liquid environment. We measured reversals and turns in animals that freely moved on a NGM agar plate that had osmolarity of ∼150 or 400 mOsm. We found that the animals on the 400 mOsm NGM plate generated significantly more turns and reversals than the animals on the 150 mOsm NGM plate within a 5 min exposure (Fig. 1*H*). Note that the rate of turns and reversals during swimming is higher than that during crawling, which is consistent with the faster locomotory dynamics previously characterized for swimming in comparison with those of crawling (Pierce-Shimomura et al., 1999; Gray et al., 2005; Korta et al., 2007; Srivastava et al., 2009; Ha et al., 2010). It has been shown that when crawling forward on a solid substrate, *C. elegans* generates sinusoidal body waves that are randomly interrupted by reorienting movements, mainly reversals and turns; attractive and aversive environmental conditions suppress or increase the reorienting movements, respectively (Pierce-Shimomura et al., 1999). Together, these results indicate that *C. elegans* responds to the upshift of osmolarity by gradually increasing reorienting maneuvers during either swimming or crawling. This navigational response takes a few minutes to occur, which is different from an acute escape-like avoidance behavior.

**Figure 2.**
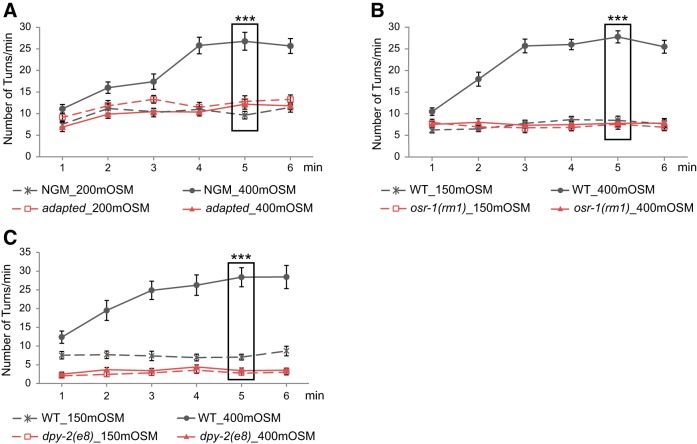
The increase in the turning rate is part of the physiologic response to hyperosmotic stresses. ***A***, Wild-type animals cultivated under the standard NGM plates of 150 mOsm display a higher turning rate in a hyperosmotic solution of 400 mOsm (NGM_400 mOsm, *n* = 32) than in a 200 mOsm solution (NGM_200 mOsm, *n* = 28); but animals that are cultivated on the plates of 400 mOsm display a turning rate in the solution of 400 mOsm (adapted_400 mOsm, *n* = 32) that is similar to that in the solution of 200 mOsm (adapted_200 mOsm, *n* = 32). Significant interaction between cultivating conditions and osmolarity is tested by two-way ANOVA. ***B***, The *osr-1(rm1)* mutant animals do not display an increased turning rate in the hyperosmotic solution of 400 mOsm (*n* = 29) compared with the turning rate in 150 mOsm (*n* = 28). Both the mutant and WT animals are cultivated under the standard osmotic condition of 150 mOsm. *n* = 29 and 31 for wild-type animals tested in solutions of 150 and 400 mOsm, respectively. ***C***, The *dpy-2(e8)* mutant animals do not display an increased turning rate in the hyperosmotic solution of 400 mOsm (*n* = 16) compared with the turning rate in 150 mOsm (*n* = 16). Both the mutant and wild-type animals are cultivated under the standard osmotic condition of 150 mOsm. *n* = 16 for WT animals tested in each condition. For ***B*** and ***C***, significant interaction between genotype and osmolarity is tested by two-way ANOVA. For all, ***p < 0.001. Values are reported as the mean ± SEM. WT, wild type.

Because maintaining a stable intracellular osmolarity is a homeostatic drive, we examined whether the aversive response to osmotic upshifts is associated with homeostatic responses. Because homeostatic responses are evoked by deviations from a baseline condition (Craig, 2003; Critchley et al., 2004), we tested animals with different internal osmolarity. Previous studies show that *C. elegans* adapts to prolonged exposure to hyperosmotic conditions by increasing the intracellular osmolarity through increased synthesis of glycerol (Lamitina et al., 2004). Thus, we predicted that adapting the animals to an external osmolarity of 400 mOsm would prevent them from increasing their turning rate in response to exposure to an osmolarity of 400 mOsm. Consistently, we found that animals that were cultivated on the NGM plate of 400 mOsm displayed similar turning rates when exposed to 400 or 200 mOsm (Fig. 2*A*). In addition, these results also show that an osmotic downshift from 400 to 200 mOsm did not induce a significant change in the turning rate. We speculate that a potential behavioral response to osmotic downshifts might be displayed with other locomotory parameters or might occur too slowly to be detected within a few minutes of exposure. We also tested *osr-1(rm1)* and *dpy-2(e8)* mutant animals. Previous studies have shown that a prolonged exposure to hyperosmotic conditions (1 Osm) decreases the body volume and reduces mobility in *C. elegans* due to the loss of water. Both the *osr-1(rm1)* and *dpy-2(e8)* mutants harbored mutations that generated resistance to the water loss induced by hyperosmotic conditions (Solomon et al., 2004; Wheeler and Thomas, 2006). We found that neither the *osr-1(rm1)* mutants nor the *dpy-2(e8)* mutants increased their turning rates in response to the osmotic upshift from 150 to 400 mOsm (Fig. 2*B*,*C*). Previous studies show that the *osr-1(rm1)* mutant animals generate normal avoidance to nose touch or the repellent 1-octanol (Solomon et al., 2004). We found that the *dpy-2(e8)* mutant animals normally avoided (WT, 85.3% ± 2.0% avoidance; *dpy-2*, 86.7% ± 1.9% avoidance, *n* = 30 animals each; *p* = 0.625, Student’s *t* test, mean ± SEM) gentle touches to the anterior part of the body (Chalfie et al., 1985). Therefore, these two mutants are not generally defective in generating avoidance response. Together, these results suggest that the aversive response to osmotic upshifts is part of the physiologic response to the hyperosmotic stress.

### The aversion to osmotic upshift requires the cGMP-gated channel subunit TAX-2

To characterize the aversive response to the osmotic upshift, we sought the underlying sensory mechanisms by examining a pool of candidate genes.

First, we examined aquaporins, which are a family of membrane proteins that are conserved throughout the animal kingdom and regulate the transport of water and small-molecule solutes, such as glycerol, cross the cell membrane (Agre et al., 2002). Eleven aquaporin homologs have been identified in *C. elegans* (Park and Saier, 1996; Ishibashi and Sasaki, 1998; Morishita et al., 2004). We tested the effect of all available mutations in *aqp-1*, *-2*, *-3*, *-4*, *-6*, *-7*, *-8*, *-9*, *-10*, and *-11* (Table 1). Among all the mutations tested, only the deletion mutation in *aqp-9* generated a significant but small reduction in the increased turning rate induced by an osmotic upshift from 150 to 400 mOsm. These results suggest that the aquaporin water channel AQP-9 may play a role in the sensorimotor response to the osmotic upshift.

**Table 1.** Channels examined for osmotic upshifts

Mutated gene	Gene family	Number of turns/min for the 5th minute				*p* value
		Wild-type control		Mutant		
		150 mOsm	400 mOsm	150 mOsm	400 mOsm	
*aqp-1*	Aquaporin	9.50 ± 1.20 (16)	27.50 ± 1.79 (16)	13.56 ± 2.48 (16)	33.80 ± 3.09 (15)	0.617
*aqp-2*	Aquaporin	10.88 ± 2.67 (8)	16.63 ± 3.68 (8)	8.00 ± 2.03 (8)	20.14 ± 4.98 (7)	0.357
*aqp-3*	Aquaporin	5.93 ± 0.91 (15)	21.87 ± 1.67 (15)	6.00 ± 1.04 (14)	16.87 ± 2.25 (15)	0.114
*aqp-4*	Aquaporin	8.44 ± 0.93 (16)	21.94 ± 1.87 (16)	9.94 ± 1.40 (16)	29.38 ± 3.15 (16)	0.146
*aqp-6*	Aquaporin	10.25 ± 1.80 (8)	28.38 ± 2.04 (8)	7.50 ± 1.38 (8)	28.13 ± 3.70 (8)	0.606
*aqp-7*	Aquaporin	10.33 ± 1.54 (12)	29.00 ± 1.62 (16)	9.19 ± 1.68 (16)	23.06 ± 1.68 (16)	0.156
*aqp-8*	Aquaporin	9.31 ± 1.76 (16)	24.63 ± 2.40 (16)	12.17 ± 1.40 (12)	27.44 ± 2.93 (16)	0.993
*aqp-9*	Aquaporin	11.5 ± 1.24 (32)	33.16 ± 1.44 (32)	11.78 ± 1.31 (32)	27.41 ± 1.35 (32)	0.026
*aqp-10*	Aquaporin	9.50 ± 1.20 (16)	27.50 ± 1.79 (16)	6.63 ± 0.80 (16)	25.67 ± 1.67 (15)	0.714
*aqp-11*	Aquaporin	8.44 ± 0.93 (16)	21.94 ± 1.87 (16)	7.88 ± 0.74 (16)	25.56 ± 2.22 (16)	0.187
*asic-1*	Epithelial sodium channel (ENaC)	13.25 ± 0.95 (36)	29.49 ± 1.47 (37)	14.74 ± 1.12 (35)	32.05 ± 1.29 (39)	0.664
*mec-4*	Epithelial sodium channel (ENaC)	16.59 ± 1.48 (32)	31.29 ± 1.58 (28)	20.11 ± 2.05 (28)	35.72 ± 1.57 (32)	0.784
*trp-4*	Transient receptor potential (TRP) channel	11.17 ± 1.02 (15)	32.29 ± 2.06 (15)	14.54 ± 1.93 (15)	35.42 ± 1.91 (15)	0.944
*tmc-1*	Transmembrane channel-like protein	14.75 ± 1.09 (24)	27.83 ± 1.89 (24)	12.29 ± 1.20 (24)	29.92 ± 1.39 (24)	0.115
*tmc-2*	Transmembrane channel-like protein	12.75 ± 1.29 (20)	28.32 ± 2.15 (22)	10.63 ± 0.98 (24)	26.09 ± 1.96 (23)	0.975
*tax-4*	Cyclic nucleotide-gated ion channel	10.25 ± 1.15 (28)	22.32 ± 1.35 (28)	11.22 ± 1.13 (27)	20.86 ± 1.12 (28)	0.309

Values are reported as the mean ± SEM (*n* animals tested). Mutants with mutations in several channel-encoding genes are examined. The number of turns per minute is shown for the 5th minute for each genotype and osmolarity treatment. The interaction of genotype and treatment is tested with two-way ANOVA on the number of turns per minute in the 5th minute. The number of animals tested under each condition is indicated in the parenthesis next to mean ± SEM.

Next, we tested the mutations in a few mechanical sensory channels, because mechanical responses are implicated in sensing osmotic stimuli (Zhang et al., 2007). We tested mutations in *asci-1* that encoded a DEG/ENaC/ASIC mechanosensory channel (Russell et al., 2014), in *trp-4* that encoded the *C. elegans* homolog of the mechanosensitive TRPN channel (Li et al., 2006), as well as in *mec-4* that encoded an amilioride-sensitive mechanosensory channel subunit (O’Hagan and Chalfie, 2006). None of the tested mutations generated a detectable defect in response to the upshift of osmolarity from 150 to 400 mOsm (Table 1). Therefore, the increased turning rate evoked by exposing to an upshift of osmolarity in *C. elegans* is likely different from the mechanosensory responses that are mediated by these channels.

**Figure 3.**
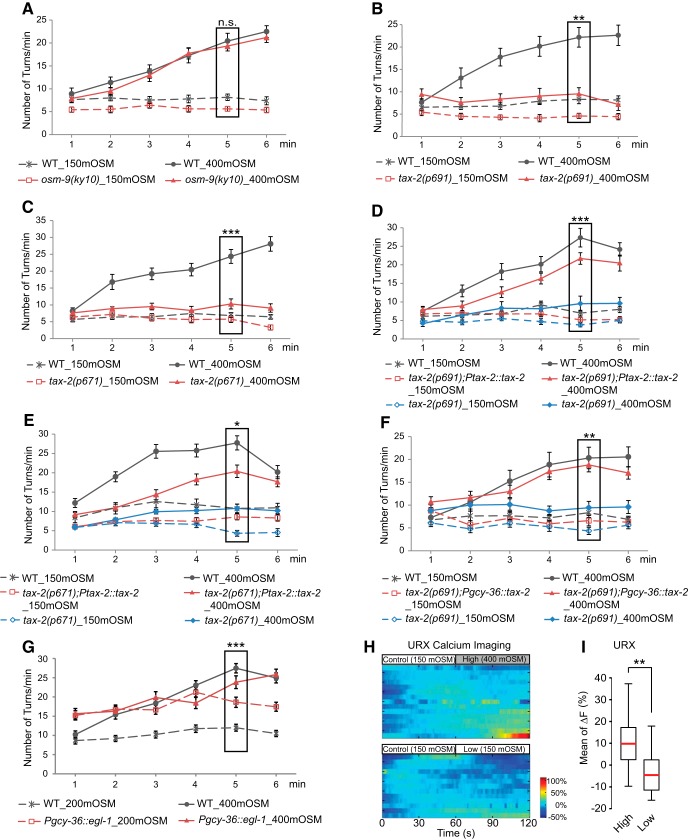
The cGMP-gated channel subunit TAX-2 acts in the AQR, PQR, and URX neurons to regulate the aversive response to osmotic upshifts. ***A***, The mutant animals *osm-9(ky10)* cultivated under the standard condition display an increased turning rate in the hyperosmotic solution of 400 mOsm that is similar to that of WT animals. *n* = 31 and 29 WT animals tested in 150 and 400 mOsm, respectively; *n* = 32 and 31 *osm-9* animals tested in 150 and 400 mOsm, respectively. ***B***, ***C***, Two mutation alleles of *tax-2*, *p691* and *p671*, are severely defective in increasing the turning rate in response to the osmotic upshift from 150 to 400 mOsm, compared with WT animals. For ***B***, *n* = 24 WT animals were tested in either 150 or 400 mOsm and *n* = 20 and 24 *p691* animals were tested in 150 and 400 mOsm, respectively; for ***C***, *n* = 23 and 24 WT animals were tested in 150 and 400 mOsm, respectively, and *n* = 21 and 27 *p671* animals were tested in 150 and 400 mOsm, respectively. ***D***, ***E***, Expressing the genomic DNA fragment containing the coding region and regulatory sequences of *tax-2* (*Ptax-2::tax-2*) rescues the defects in both *tax-2* alleles. For ***D***, *n* = 32 and 31 WT animals tested in 150 and 400 mOsm, respectively; *n* = 29 and 31 transgenic animals expressing *Ptax-2::tax-2* tested in 150 and 400 mOsm, respectively; and *n* = 28 and 26 *p691* mutant sibling animals tested in 150 and 400 mOsm, respectively; for ***E***, *n* = 31 and 32 WT animals were tested in 150 and 400 mOsm, respectively; *n* = 31 and 32 transgenic animals expressing *Ptax-2::tax-2* were tested in 150 and 400 mOsm, respectively; and *n* = 30 *p671* mutant sibling animals were tested in either 150 or 400 mOsm. ***F***, Expressing the wild-type *tax-2* cDNA in the body cavity sensory neurons AQR, PQR, URX (*Pgcy-36::tax-2*) rescues the defects in the *tax-2(p691)* mutants in generating an increased turning rate in response to the osmotic upshift from 150 to 400 mOsm. *n* = 24 and 22 WT animals tested in 150 and 400 mOsm, respectively, *n* = 22 and 24 transgenic animals expressing *Pgcy-36::tax-2* tested in 150 and 400 mOsm, respectively, *n* = 23 and 24 *p691* mutant sibling animals tested in 150 and 400 mOsm, respectively. ***G***, Expressing cell death-promoting molecule EGL-1 in the body cavity sensory neurons AQR, PQR, and URX (*Pgcy-36::egl-1*) abolished the increased turning rate in response to the osmotic upshift from 200 to 400 mOsm. *n* = 36 and 44 WT animals were tested in 200 and 400 mOsm, respectively; *n* = 40 and 44 transgenic animals expressing *Pgcy-36::eg-1* were tested in 200 and 400 mOsm, respectively. ***H***, The heat map of the calcium signals in the URX neuron in individual animals that were exposed to 150 mOsm and switched to 400 mOsm and in individual animals that were exposed to 150 mOsm and switched to 150 mOsm. The calcium signal was presented as the percentage change in GCaMP6 signal using the average GCaMP6 signal in the first 60 s as the baseline [(*F* − *F*_base_)/*F*_base_ * 100%; see Materials and Methods]. ***I***, The average change in the GCaMP6 signal in URX is significantly higher when animals are switched from 150 to 400 mOsm (*n* = 15) than when animals are switched from 150 to 150 mOsm (*n* = 13). Box plot shows the first and third quartile, median, and the maximum and minimum. The Student’s *t* test was used for comparison. For ***A–C*** and ***G***, significant interaction between genotype (wild-type vs mutant animals) and osmolarity is tested with two-way ANOVA; for ***D–F***, significant interaction between genotype (transgenic vs nontransgenic siblings) and osmolarity is tested with two-way ANOVA. For all, animals are cultivated under the standard NGM plates with an osmotic condition of 150 mOsm. ***p < 0.001, **p < 0.01, *p < 0.05. n.s., Not significant. Values are reported as the mean ± SEM. WT, wild type.

To further explore the neuronal mechanisms underlying the sensory response of osmotic upshifts, we examined mutations in channels that were known to regulate responses to different chemosensory cues. Previous studies have shown that *C. elegans* acutely avoids solutions of a high concentration of sodium and generates reversals in a few seconds after a direct contact with the salt. This response requires the function of a sodium-sensitive channel transmembrane channel-like 1 (TMC-1) in the polymodal sensory neurons ASH (Chatzigeorgiou et al., 2013). A recent study shows that TMC-1 regulates an acute avoidance of alkaline pH in ASH (Wang et al., 2016). *C. elegans* generates a similar acute avoidance of osmotic shocks produced by solutions of high osmolarity, such as 1 m glycerol or 4 m fructose, and the avoidance depends on the function of a *C. elegans* TRPV channel, OSM-9, in the ASH sensory neurons (Colbert et al., 1997; Liedtke et al., 2003; Hilliard et al., 2004). We found that mutations in *osm-9*, *tmc-1*, or *tmc-2*, which encoded a homolog of *tmc-1,* did not disrupt the aversive behavioral response to the osmotic upshift (Fig. 3*A*, Table 1). In contrast, two canonical mutations, *p691* and *p671*, in *tax-2* that encoded a cGMP-gated channel subunit and was known to regulate multiple sensory responses, including olfaction (Bargmann, 2006), severely disrupted the increased turning rate in response to the osmotic upshift from 150 to 400 mOsm (Fig. 3*B*,*C*). The defects in both alleles were rescued by a genomic fragment that contained the regulatory and the coding regions of *tax-2* (Fig. 3*D*,*E*). These results indicate that the cGMP-gated channel subunit TAX-2 is required for the sensorimotor response to the osmotic upshift and that the gradually generated aversive response to the osmotic upshift defines a new sensory-evoked behavior that is different from the avoidance of a high salt solution or osmotic shocks.

### TAX-2 regulates osmotic response in the sensory neurons that are exposed to the body fluid

Several sensory neurons in *C. elegans* mediate sensorimotor responses through the cGMP-gated channel TAX-2/TAX-4 (Bargmann, 2006). Next, we asked in which sensory neurons *tax-2* regulated the aversive response to osmotic upshifts.

First, we tested the effect of several mutations that generated defects in the development of subsets of the *tax-2-*expressing sensory neurons. We tested the *ttx-1(p676)* and the *che-1(p679)* mutants, which were defective in the development and differentiation of the sensory neurons AFD and ASE, respectively (Satterlee et al., 2001; Uchida et al., 2003). We also examined the transgenic animals that expressed a gain-of-function isoform of potassium channel *twk-18* in the sensory neuron ADF [*Psrh-142::twk-18(gf)*] (Qin et al., 2013). The expression of *twk-18(gf)* hyperpolarizes and inhibits the activity of the expressing neurons (Kunkel et al., 2000). None of the transgenic animals or the mutant animals exhibited a detectable defect in their response to the osmotic upshift (Table 2). These results suggest that the sensory neurons ADF, AFD, or ASE are not required for the increased turning rate evoked by the upshift of osmolarity from 150 to 400 mOsm.

**Table 2.** Sensory neurons examined for osmotic upshifts

Mutated gene/transgene	Affected neurons	Number of turns/min for the 5th minute				*p* value
		Wild-type control		Mutant/transgenic		
		200# mOsm	400 mOsm	200# mOsm	400 mOsm	
*ttx-1*	AFD	10.40 ± 0.79 (30)	22.20 ± 2.35 (20)	9.86 ± 0.80 (28)	21.15 ± 1.86 (27)	0.863
*che-1*	ASE	14.36 ± 1.21 (28)	30.31 ± 1.72 (32)	18.19 ± 1.62 (32)	28.28 ± 1.41(32)	0.057
*Psrh-142::twk-18*	ADF	6.75 ± 0.73 (8)	26.00 ± 2.41 (8)	8.57 ± 1.65 (7)	24.25 ± 1.47 (8)	0.298

Values are reported as the mean ± SEM (*n* animals tested). Mutants defective in the development and/or function of several sensory neurons are examined for osmotic upshifts. *ttx-1* and *che-1*, as well as the wild-type controls for these two mutants, are tested at 200 and 400 mOsm for osmotic upshifts. The transgenic animals that express *Psrh-142::twk-18(gf)* and the nontransgenic siblings are tested at 150 and 400 mOsm for osmotic upshifts. The number of turns per minute is shown for the 5th minute for each genotype and osmolarity treatment. The interaction of genotype and treatment is tested with two-way ANOVA on the number of turns per minute in the 5th minute. The number of animals tested under each condition is indicated in the parenthesis next to mean ± SEM.

# *ttx-1* and *che-1*, as well as the wild-type controls for these two mutants, were tested at 200 and 400 mOsm for osmotic upshifts. The transgenic animals that express *Psrh-142::twk-18(gf)* and the nontransgenic siblings are tested at 150 and 400 mOsm for osmotic upshifts.

Next, to identify the *tax-2-*expressing sensory neurons that regulate the aversive response to the osmotic upshift, we expressed the wild-type *tax-2* cDNA selectively in a few sensory neurons in the *tax-2(p691)* mutants and examined the potential rescuing effects. Because we found that exposure to the osmotic upshift from 150 to 400 mOsm for a few minutes generated a significant shrinkage of the worm body, we hypothesized that osmotic upshifts might alter the osmolarity of the body fluid. Thus, we expressed *tax-2* in AQR, PQR, and URX (*Pgcy-36::tax-2*), which comprised a set of sensory neurons that were exposed to the body cavity. We found that the expression of *tax-2* in AQR, PQR, and URX fully rescued the defect of the *tax-2(p691)* mutant animals in responding to the osmotic upshift from 150 to 400 mOsm (Fig. 3*F*). In contrast, expressing *tax-2* in the main olfactory sensory neurons AWB and AWC using the *odr-1* promoter (*Podr-1::tax-2*; Qin et al., 2013) or in the sensory neuron ASI (*Pstr-3::tax-2*) had no detectable rescuing effect in the aversive behavioral response (Table 3). These results together show that the function of TAX-2 in the body cavity neurons are sufficient to regulate the aversive response to the osmotic upshift. The ciliated endings or the somata of the AQR, PQR, and URX neurons are directly exposed to the body fluid and are referred to as the body cavity sensory neurons (White et al., 1986). To further examine the role of the body cavity neurons, we tested the transgenic animals that expressed the cell death-promoting molecule EGL-1 (Conradt and Horvitz, 1998) selectively in these neurons (Chang et al., 2006). We found that the transgenic animals displayed a behavioral response significantly different from that of wild-type animals. The animals without AQR, PQR, and URX generated a higher turning rate at 200 mOsm and did not significantly increase the turning rate on exposure to 400 mOsm (Fig. 3*G*). These results suggest that the body cavity neurons regulate the turning rate at 200 mOsm, which may prevent a further increase in the turning rated at the higher osmolarity. Nevertheless, the result reveals an important role of the body cavity neurons in regulating the turning rate under either the standard or higher osmotic condition or both. Together, these results indicate that the body cavity sensory neurons mediate the aversive response to the osmotic upshift via the function of the cGMP-gated channel subunit TAX-2.

**Table 3.** Cell-specific rescue of *tax-2* mutants examined for osmotic upshifts

Genetic background	Transgene	Expressing neurons	Number of turns/min for the 5th minute Mean ± SEM (n)				*p* value
			Nontransgenic control		Transgenic animals		
			150 mOsm	400 mOsm	150 mOsm	400 mOsm	
*tax-2(p691)*	*Podr-1::tax-2*	AWB, AWC	5.66 ± 0.69 (29)	9.33 ± 0.89 (30)	7.63 ± 1.15 (27)	9.86 ± 1.11 (28)	0.560
*tax-2(p691)*	*Pstr-3::tax-2*	ASI	6.84 ± 0.61 (31)	12.31 ± 1.25 (32)	8.55 ± 0.83 (31)	17.14 ± 2.04 (29)	0.565

Values are reported as the mean ± SEM (*n* animals tested). Transgenic animals that express a wild-type *tax-2* cDNA in the major olfactory sensory neurons AWB and AWC (*Podr-1::tax-2*) or in the sensory neuron ASI (*Pstr-3::tax-2*) in the *tax-2(p691)* mutant background are examined for osmotic upshifts. The number of turns per minute is shown for the 5th minute for each genotype (transgenic vs nontransgenic siblings) and osmolarity treatment. The interaction of genotype and treatment is tested with two-way ANOVA on the number of turns per minute in the 5th minute. The number of animals tested under each condition is indicated in the parenthesis next to mean ± SEM.

To characterize the mechanisms underlying the function of the body cavity sensory neurons in mediating the aversive response to the osmotic upshift, we examined the intracellular calcium transients in the sensory neuron URX under the standard condition and during the osmotic upshift. We focused on the URX neuron because the function of URX in regulating the sensorimotor response to another environmental cue, oxygen, has been well characterized (Cheung et al., 2004; Gray et al., 2004; Zimmer et al., 2009; Witham et al., 2016). We used the transgenic lines that expressed a genetically encoded fluorescent reporter, GCaMP6 (Chen et al., 2013), in URX and a microfluidic device (Chronis et al., 2007) that allowed the head of the transgenic animal exposed to a stream of solution. The solution could be switched between two alternatives that were controlled by an air flow. We first exposed the transgenic animal to a solution of 150 mOsm for 60 s and then switched the stimulating fluidic stream to a solution of either 150 or 400 mOsm. We recorded the GCaMP6 signal in the cell body of the URX neuron and quantified the change of GCaMP6 signals in response to the switch of the solution (Materials and Methods). We found that URX generated calcium signals with higher amplitudes during the exposure to 400 mOsm than during the exposure to 150 mOsm (Fig. 3*H*,*I*). Although the increase in the URX calcium signal is moderate, it is significant. Previous studies show that URX responds to increased environmental oxygen concentrations by transiently increasing the intracellular calcium signals (Cheung et al., 2004; Gray et al., 2004; Zimmer et al., 2009; Witham et al., 2016) and that ectopically activating URX evokes aversive behavior (Busch et al., 2012). Together, these results demonstrate that the osmotic upshift from 150 to 400 mOsm activates the body cavity neuron URX to mediate the aversive behavioral response.

Previous studies have shown that guanylyl cyclases act downstream of G-protein-coupled receptors to produce cGMP that regulate the function of the cGMP-gated TAX-2/TAX-4 channels (Bargmann, 2006). Next, we examined the Gα subunits and the guanylyl cyclases that were expressed in the AQR, PQR, and URX neurons. The *C. elegans* genome encodes a large number of Gα proteins, GPA-1 to GPA-16, ODR-3, GOA-1, and ELG-30 (Jansen et al., 1999). We tested mutations in *gpa-8* and *gpa-13* that were expressed in AQR, PQR, and URX (Table 4). We found that neither of the mutations generated a significant defect in responding to the osmotic upshift. These results suggest that these Gα proteins are not required for the osmotic response or that they act in a redundant manner. We also examined mutations in the genes that encoded guanylyl cyclases and were shown to be expressed in the AQR, PQR, and URX neurons (Table 4). The pool included *gcy-28*, which encoded a receptor guanylyl cyclase and regulated an innate olfactory preference and integration (Tsunozaki et al., 2008; Shinkai et al., 2011); *gcy-33, 35* and *gcy-36*, all of which encoded soluble guanylyl cyclases that mediated responses to oxygen (Cheung et al., 2004; Gray et al., 2004); as well as several other guanylyl cyclases whose functions were less well characterized (Yu et al., 1997; Ortiz et al., 2006). Again, we found that none of the mutations significantly altered the behavioral response to the osmotic upshift (Table 4). These results show that the tested guanylyl cyclases are not required for the response to the osmotic upshift or they act redundantly.

**Table 4.** G-Protein pathway components examined for osmotic upshifts

Mutated gene	Expressing neurons+	Number of turns/min for the 5th minute				*p* value
		Wild-type control		Mutant		
		150 mOsm	400 mOsm	150 mOsm	400 mOsm	
*gpa-8*	AQR, PQR, URX	9.58 ± 1.02 (24)	24.61 ± 1.51 (23)	7.33 ± 0.85 (21)	20.67 ± 1.89 (21)	0.538
*gpa-13*	PQR	7.19 ± 1.13 (16)	16.13 ± 2.91 (15)	9.63 ± 2.23 (16)	21.69 ± 1.98 (16)	0.467
*gcy-1*	URX	8.19 ± 1.50 (16)	29.00 ± 1.91 (16)	5.88 ± 1.01 (16)	25.31 ± 2.22 (16)	0.691
*gcy-25*	AQR, PQR, URX	11.07 ± 1.52 (15)	26.73 ± 2.10 (15)	8.50 ± 1.10 (16)	23.13 ± 1.26 (16)	0.734
*gcy-28*	Widely expressed	9.31 ± 1.76 (16)	24.63 ± 2.40 (16)	9.00 ± 1.14 (16)	32.06 ± 2.18 (16)	0.049#
*gcy-32*	AQR, PQR, URX	11.06 ± 1.80 (16)	27.60 ± 1.77 (15)	7.71 ± 0.71 (14)	25.81 ± 1.61 (16)	0.622
*gcy-33*	AQR, PQR, URX	10.94 ± 2.24 (16)	22.88 ± 2.67 (16)	8.88 ± 1.13 (16)	18.87 ± 2.34 (15)	0.655
*gcy-34*	AQR, PQR, URX	7.33 ± 0.84 (15)	22.94 ± 2.14 (16)	7.73 ± 1.01 (15)	20.19 ± 2.37 (16)	0.377
*gcy-35*	AQR, PQR, URX	9.83 ± 1.39 (23)	30.08 ± 1.25 (24)	9.68 ± 1.26 (22)	28.46 ± 1.40 (24)	0.578
*gcy-36*	AQR, PQR, URX	9.83 ± 1.39 (23)	30.08 ± 1.25 (24)	8.42 ± 1.18 (24)	27.39 ± 1.99 (23)	0.665
*gcy-37*	AQR, PQR, URX	9.94 ± 1.25 (16)	26.69 ± 2.15 (16)	6.93 ± 0.73 (15)	21.87 ± 2.14 (15)	0.592

Values are reported as the mean ± SEM (*n* animals tested). Mutants that are mutated for genes that encode G-protein pathway components and are expressed in AQR, PQR, and URX are examined. The number of turns per minute is shown for the 5th minute for each genotype and osmolarity treatment. The interaction of genotype and treatment is tested with two-way ANOVA on the number of turns per minute in the 5th minute. The number of animals tested under each condition is indicated in the parenthesis next to mean ± SEM.

+ Only the expression in AQR, PQR or URX neurons is indicated.

# The rate of turns increases more in the mutant than in the wild-type control.

### The neural circuit underlying navigational locomotion mediates the behavioral aversion to osmotic upshifts

**Figure 4.**
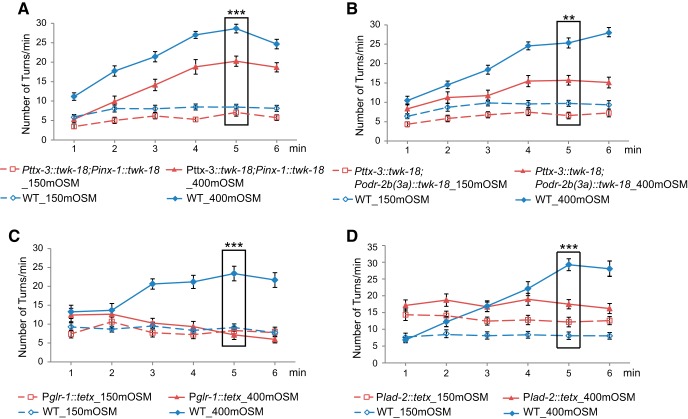
The neural circuit underlying navigation regulates the aversive response to osmotic upshifts. ***A***, Inhibiting the activity of the interneurons AIB and AIY by expressing *twk-18(gf)* produces significant defects in generating an increased turning rate in response to the osmotic upshift from 150 to 400 mOsm. *n* = 40 and 38 transgenic animals expressing *twk-18(gf)* in AIB and AIY were tested in 150 and 400 mOsm, respectively; and *n* = 40 and 38 WT sibling animals were tested in 150 and 400 mOsm, respectively. ***B***, Inhibiting AIB, AIY, and AIZ by expressing *twk-18(gf)* produces significant defects in generating an increased turning rate in response to the osmotic upshift from 150 to 400 mOsm. *n* = 40 and 33 transgenic animals expressing *twk-18(gf)* in AIB, AIY, and AIZ were tested in 150 and 400 mOsm, respectively; *n* = 40 and 39 WT sibling animals were tested in 150 and 400 mOsm, respectively. ***C***, Blocking the neural transmission of several command interneurons and motor neurons by expressing the tetanus toxin with the *glr-1* promoter produces significant defects in generating an increased turning rate in response to the osmotic upshift from 150 to 400 mOsm. *n* = 24 animals in all conditions. ***D***, Blocking the neural transmission of several motor neurons by expressing the tetanus toxin with the *lad-2* promoter produces significant defects in generating an increased turning rate in response to the osmotic upshift from 150 to 400 mOsm. *n* = 23 transgenic animals expressing the tetanus toxin with the *lad-2* promoter were tested in either 150 or 400 mOsm; and *n* = 22 and 23 WT sibling animals were tested in 150 and 400 mOsm, respectively. For all, animals are cultivated on the standard NGM plates with an osmotic condition of 150 mOsm, and the significant interaction between genotype (transgenic vs nontransgenic siblings) and osmolarity is tested using two-way ANOVA, ***p < 0.001, **p < 0.01. Values are reported as the mean ± SEM. WT, wild type.

To further understand how the osmotic upshift generates an aversive behavioral response, we probed the downstream neural circuit. The interneurons AIB, AIY, and AIZ are the first layer of interneurons that connect the sensory input with the downstream motor system (White et al., 1986). To test the potential role of these interneurons, we expressed the gain-of-function isoform of the potassium channel *twk-18(gf)* (Kunkel et al., 2000) to inhibit the activity of all or subsets of these interneurons (Luo et al., 2014) and examined the resulting effect on behavior. We found that expressing *twk-18(gf)* in either AIY alone [*Pttx-3::twk-18(gf)*] or primarily in AIB [*Pinx-1::twk-18(gf)*] generated no obvious phenotype in the aversive response evoked by the osmotic upshift (Table 5). In contrast, combining the two transgenes [Fig. 4*A*, *Pttx-3::twk-18(gf); Pinx-1::twk-18(gf)*] significantly reduced the increase in the turning rate in response to the osmotic shift from 150 to 400 mOsm. In addition, expressing *twk-18* in AIB, AIY, and AIZ together [Fig. 4*B*, *Pttx-3::twk-18(gf); Podr-2b(3a)::twk-18(gf)*] similarly suppressed the aversive behavior, but expressing *twk-18* mainly in AIB and AIZ [*Podr-2b(3a)::twk-18(gf)*] was not sufficient to generate a detectable defect (Table 5). Together, these results show that the interneurons AIB and AIY together play a significant role in mediating the behavioral aversion to the osmotic upshift.

**Table 5.** Interneurons and motor neurons examined for osmotic upshifts

Transgene	Affected neurons	Number of turns/min for the 5th minute				*p* value
		Nontransgenic control		Transgenic worms		
		150 mOsm	400 mOsm	150 mOsm	400 mOsm	
*Podr-2b(3a)::twk-18(gf)*	AIB, AIZ	9.38 ± 1.19 (16)	25.69 ± 1.96 (16)	5.63 ± 1.38 (16)	22.60 ± 2.24 (15)	0.848
*Pinx-1::twk-18(gf)*	AIB	7.44 ± 1.19 (16)	29.31 ± 2.22 (16)	1.63 ± 0.51 (16)	26.56 ± 2.04 (16)	0.355
*Pttx-3::twk-18(gf)*	AIY	7.81 ± 0.99 (16)	25.06 ± 2.26 (16)	7.56 ± 1.08 (16)	23.40 ± 1.92 (15)	0.669

Values are reported as the mean ± SEM (*n* animals tested). Transgenic animals that express a gain-of-function isoform of the potassium channel TWK-18 to inhibit the activity of specific interneurons and/or motor neurons are examined. The number of turns per minute is shown for the 5th minute for each genotype (transgenic vs nontransgenic siblings) and osmolarity treatment. The interaction of genotype and treatment is tested with two-way ANOVA on the number of turns per minute in the 5th minute. The number of animals tested under each condition is indicated in the parenthesis next to mean ± SEM.

Previous findings show that AIB and AIY interneurons act upstream of several command interneurons and motor neurons to regulate reversals and turns (Tsalik and Hobert, 2003; Gray et al., 2005; Ha et al., 2010). Thus, we further examined the locomotory circuit. We found that expressing the tetanus toxin (Schiavo et al., 1992) with the *glr-1* promoter to block presynaptic release from several command interneurons and motor neurons (Hendricks et al., 2012) completely abolished the increase in the turning rate induced by the osmotic shift (Fig. 4*C*). The *glr-1* promoter is expressed in several command interneurons, including AVA, which is known to regulate reversals, and a few motor neurons, such as SMD and RIM that regulate head bending and turns during escape responses (Chalfie et al., 1985; Brockie et al., 2001; Gray et al., 2005; Ha et al., 2010). We also used the *lad-2* promoter (Wang et al., 2008) to block neurotransmission from several motor neurons that regulate head bending, including SMD (Hendricks et al., 2012). We found that the mutation also generated a significant defect in the osmotic upshift-induced increase in the turning rate (Fig. 4*D*). Together, these results indicate that the aversive response to the osmotic upshift uses the neural circuit that underlies the head bendings and turns to regulate navigational locomotion.

## Discussion

Under the standard laboratory conditions, the nematode *C. elegans* lives in an environment with a relatively constant osmolarity; however, in its natural habitat of rotten organic materials, changes in osmolarity are commonplace. Because both hyperosmotic and hypoosmotic conditions generate adverse effects on cellular functions, it is critical that animals use behavioral and physiologic responses to reduce the osmotic stresses. *C. elegans* is known to acutely avoid extremely high osmolarity (≥1 Osm) via the OSM-9 TRPV channel, which acts in the polymodal sensory neuron ASH, which is open to the outside (Colbert et al., 1997; Liedtke et al., 2003). Here, we show that *C. elegans* also generates an aversive behavioral response when it is continuously exposed to the upshift of osmolarity for a couple minutes. The locomotory response evoked by the osmotic upshift is characterized by a gradual increase in the frequency of turns and reversals. In addition, this slow behavioral response is regulated by the function of a cGMP-gated channel subunit TAX-2 in a set of sensory neurons, AQR, PQR, and URX, that are exposed to the body fluid. Osmotic upshifts activate URX, which is known to generate aversive behavioral responses on activation. The aversive response to the osmotic upshift requires a motor circuit that underlies navigational locomotion. Together, our findings characterize a new type of aversive behavioral response that *C. elegans* uses to cope with an environmental stress, osmotic upshifts; and to identify the underlying molecular, cellular, and circuit mechanisms.

On changes in the extracellular osmolarity, animals use both homeostatic responses and behavioral strategies to alleviate the osmotic stress. It is known that a hyperosmotic condition can dehydrate *C. elegans,* resulting in death (Solomon et al., 2004). During a prolonged exposure to a hyperosmotic environment, *C. elegans* increases the production of glycerol, which increases the intracellular osmolarity and prevents water loss (Lamitina et al., 2004; Solomon et al., 2004; Wheeler and Thomas, 2006). Here, we show that an upshift of the environmental osmolarity also evokes a gradual increase in the rate of reversals and turns, a reorienting behavior that the animal generates under adverse conditions. This navigational response may be similar to the osmotaxis behavior described in bacterial *Escherichia coli* (Li et al., 1988). Meanwhile, on encountering a solution with an extremely high osmolarity, the nematode acutely reverses from the stimulus and moves away within a few seconds (Colbert et al., 1997; Liedtke et al., 2003). The combination of these behavioral and physiologic strategies together protects the nematode from the physiologic stresses generated by different hyperosmotic conditions.

The gradual increase in the turning rate in response to the osmotic upshift requires the cGMP-gated channel subunit TAX-2, which is known to regulate chemosensory responses downstream of the G-protein and cGMP signaling pathways (Bargmann, 2006). However, none of the tested Gα and guanylyl cyclases (Table 4) are required for the osmotic upshift-evoked increase in the turning rate. In addition, *tax-4*, which also encodes a cyclic nucleotide-gated channel subunit and regulates many sensory responses to chemicals, is dispensable for the aversive response to the osmotic upshifts (Table 1). The differential requirement of TAX-2 versus TAX-4 in the hyperosmotic response is similar to that in the light-evoked avoidance that depends on TAX-2, but not TAX-4 (Ward et al., 2008). These results together suggest that the mechanisms underlying the role of TAX-2 in the hyperosmotic behavioral response may be different from that in the chemical responses. It is known that hyperosmotic conditions can alter the intracellular concentration of different molecules due to water loss, which may possibly underlie the regulation of the activity of TAX-2 during osmotic upshifts. Note that the identification of TAX-2 results from testing a selected pool of channels, which does not exclude a potential role of other channels in regulating the aversive response induced by osmotic upshifts.

We also show that TAX-2 acts in a set of sensory neurons, AQR, PQR, and URX, to regulate the increased turning rate in response to the osmotic upshift. The AQR, PQR, and URX neurons are exposed to the body cavity (White et al., 1986). These anatomic characteristics may allow these neurons to sense the hyperosmotic conditions. Previous studies show that *C. elegans* prefers a range of the ambient oxygen concentrations and that the AQR, PQR, and URX neurons contribute to the regulation of the behavioral preference. The oxygen response of URX is modulated by the fat storage of the animal (Witham et al., 2016), and URX also plays an important role in regulating fat loss, body length, and life span (Mok et al., 2011; Liu and Cai, 2013; Noble et al., 2013). These findings suggest the role of the body cavity neurons in coordinating the internal and external environments with the behavioral and physiologic response of the animal. Interestingly, the guanylyl cyclases GCY-35 and GCY-36 are required for URX to respond to changes in the oxygen concentration (Gray et al., 2004; Cheung et al., 2005; Zimmer et al., 2009; Witham et al., 2016); however, mutations in either *gcy-35* or *gcy-36* or several other guanylyl cyclases that are known to express in AQR, PQR, or URX do not disrupt the behavioral response to the osmotic upshift. While URX is able to respond to both an increase in the environmental oxygen concentration and an increase in the environmental osmolarity, the neuronal response, measured by genetically encoded calcium reporters, that is evoked by the osmotic upshift appears to be much milder than that evoked by the oxygen upshift (Fig. 3; Zimmer et al., 2009; Busch et al., 2012; Witham et al., 2016). The different levels of the intracellular calcium responses under these two conditions may contribute to the different signaling pathways that are used to mediate the downstream responses. Intriguingly, while both the body cavity sensory neurons and the interneurons AIB and AIY are important for the hyperosmotic response, these two groups of neurons are not directly connected via either chemical or electrical synapses (White et al., 1986). Therefore, the AIB and AIY interneurons may interact with the body cavity neurons via nonsynaptic signals. Alternatively, these interneurons may regulate the hyperosmotic response by modulating the function of the body cavity neurons. These findings together suggest that while the body cavity neurons can mediate responses to different internal and external cues, they use distinct signaling transduction pathways to generate coherent behavioral outputs in a complex environment.

## Synthesis

Reviewing Editor: Anna Menini, SISSA (International School of Advances Studies) Decisions are customarily a result of the Reviewing Editor and the peer reviewers coming together and discussing their recommendations until a consensus is reached. When revisions are invited, a fact-based synthesis statement explaining their decision and outlining what is needed to prepare a revision will be listed below. The following reviewer(s) agreed to reveal their identity: Jill Bettinger, William Ryu

Both reviewers agree that this manuscript describes a newly identified response to osmolarity changes in the external environment, it is interesting and worthwhile of publication in eNeuro. There are mainly minor issues to be addressed that require only to be reviewed by the editor.

Both reviewers have identified the same problem with the calcium imaging results, as the statistical significance appear to be driven by one trace only. Authors should address this problem by either doing more analysis, or by making clear in the text the limitations of this experiment.

Please also address the other issues listed below.

Reviewer #1

Please put the number of calcium traces done for this work in the manuscript. n= what?

Please address in the discussion that the calcium imaging significance is driven by one trace, making it clear to a reader to be careful with interpretation of this result.

I still have two major concerns with this work, which diminishes my enthusiasm for it. However, I think that they do not make it unpublishable, so require no further addressing in this manuscript:

1- it doesn't make intellectual sense that the animals respond to upshift but not downshift of osmolarity, especially since they can _adapt_ to their culture osmolarity.

2- the calcium imaging results are simply not convincing. It looks like the significance is driven by a single trace. One result is just not scientifically rigorous. I agree that the result is statistically significant, but it is still not convincing.

Reviewer #2

The results on the changes in turn rate due to osmotic changes are important and should be published. They broaden what is known about the behavioral response to changes in osmolarity an important environmental parameter, and these changes have broad implications on C. elegans navigation. I generally agree with what is reported in the abstract, but I have concerns about the interpretation of some of the secondary results.

1) The WT data seems to vary quite a bit from trial to trial (e.g. Table 1). Can you please include more information about how the WT control data is taken? Are WT and comparative data sets taken on the same slide (e.g. 2 droplets are control and 2 are comparative tests?)

2) I'm not convinced by the GCamp URX data. There is clearly one major outlier in the 400 mOSM data set with 100% increase in signal with all other signals seem to be much less than 50%. What happens if this outlier is removed? Is the student t-test here appropriate (i.e. data are normally distributed?)

3) Fig 2. I believe some positive controls for osr-1 avoidance behavior have been reported (Solomon et. al. 2004). How about for dpy-2? I would guess they do not have WT locomotion and they are showing very low basal turning rates. This worries me a bit and so I would feel better if I could see a positive control here for some avoidance behavior.

Minor comments:

1) It should be mentioned that the shrinkage due to dehydration was measured as change in AREA of the worm image. I realize it is in the M&M, but when you read shrank 77.6% you immediately ask “in length? Volume?”. Why not answer right in the text?

2) I don't think “escape” is the right term in the sub-heading: “The neural circuit underlying escape mediates the behavior aversion to osmotic upshift.” Escape to me suggests the stereotyped response to a noxious stimuli. The neurons here are more general to locomotion and navigation.

3) It might be helpful to a more general reader if you defined the differences between “acute avoidance” and “aversion” more clearly. The response to the osmotic drop assay is a stereotyped escape response, involving a series of reversals (and often ending with an omega turn). The turn behavior you are measuring here is more of a tactic or navigational response.

4) Following up on point 3, the change in turning rate here suggests the ability for the worm to
